# Prolapsed rectal submucosal hematoma in pediatric case

**DOI:** 10.11604/pamj.2020.37.154.26149

**Published:** 2020-10-13

**Authors:** Kiran Khandare, Pradnya Ghormode

**Affiliations:** 1 Mahatma Gandhi Ayurved College Hospital and Research Center, Salod (H), Wardha, Maharashtra, India,; 2 Dayabhai Maoji Majithiya Ayurved Mahavidyalaya, Yavatmal Maharashtra, India

**Keywords:** Hematoma, rectal, submucosal

## Image in medicine

A rectal submucosal hematoma, imperfectly called a thrombosed external pile or external hemorrhoid, is a collection of blood within the skin around the anus, occurring due to a ruptured blood vessel within the submucosa usually caused by some minor trauma such as hard stool or due to severe constipation. Present case is a six year old girl child came into view with large mass coming out from anus with red dark-purple collection, pain and discomfort. She was unable to walk or sit. On examination no significant history of trauma except constipation, there was no history of per-rectal bleeding. Suddenly a swelling appeared after defecation. She was hospitalized for surgical excision. After hospitalization this swelling burst and reduced with discharge of clotted blood.

**Figure 1:**
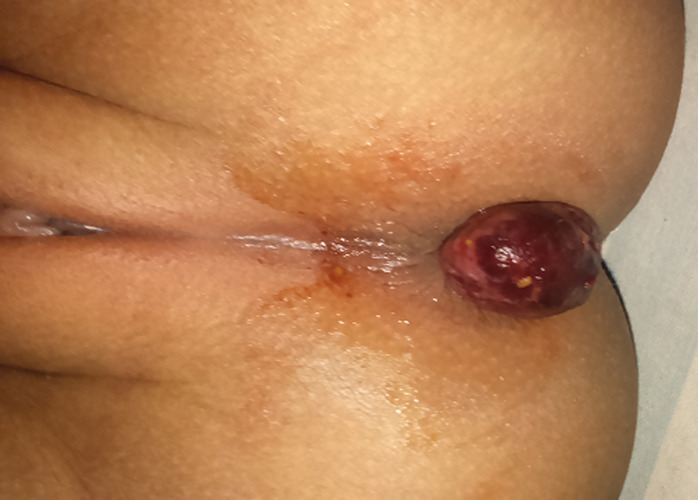
prolapsed rectal submucosal hematoma in pediatric case

